# A Thermal Model for Carbon Nanotube Interconnects

**DOI:** 10.3390/nano3020229

**Published:** 2013-04-26

**Authors:** Kaji Muhammad Mohsin, Ashok Srivastava, Ashwani K. Sharma, Clay Mayberry

**Affiliations:** 1 School of Electrical Engineering and Computer Science, Louisiana State University, Baton Rouge, LA 70803, USA; E-Mail: kmohsi1@lsu.edu; 2 Air Force Research Laboratory/Space Electronics Branch, Space Vehicles Directorate, Electronics Foundations Group, 3550 Aberdeen Avenue SE, Kirtland, NM 87117, USA; E-Mails: ashwani.sharma@kirtland.af.mil (A.K.S.); clay.mayberry@kirtland.af.mil (C.M.)

**Keywords:** SWCNT, VLSI interconnect, Joule heating, scattering

## Abstract

In this work, we have studied Joule heating in carbon nanotube based very large scale integration (VLSI) interconnects and incorporated Joule heating influenced scattering in our previously developed current transport model. The theoretical model explains breakdown in carbon nanotube resistance which limits the current density. We have also studied scattering parameters of carbon nanotube (CNT) interconnects and compared with the earlier work. For 1 µm length single-wall carbon nanotube, 3 dB frequency in S_12_ parameter reduces to ~120 GHz from 1 THz considering Joule heating. It has been found that bias voltage has little effect on scattering parameters, while length has very strong effect on scattering parameters.

## 1. Introduction

The current complementary metal oxide semiconductor (CMOS) technology in nm- and sub-nm node for very large scale integration (VLSI) is facing challenges due to performance limitation of Cu/low-k dielectric material as an interconnection, because of increased resistivity of Cu, electromigartion and void formation [1,2]. Many alternatives to Cu interconnection in nanometer technology node have been proposed in the literature, including optical interconnects. The 1D carbon nanotubes and 2D graphene nanoribbons are found to be very promising alternatives to current Cu interconnects for use in nm-CMOS technology [3]. Early discovery of carbon nanotubes in 1991 by Iijima [4] and its excellent electrical, mechanical and thermal properties and established synthesis techniques [5,6,7,8,9], have led to major R&D efforts in integrating carbon nanotubes in CMOS processes [6,7,8,9,10], whereas graphene nanoribbon as a possible substitute for Cu interconnect is also evolving [11].

Though carbon nanotube has high thermal conductivity, it has been observed experimentally that the conducting carbon nanotube breaks down due to Joule heating which thus limits its current density [11,12]. Notable work has been done in electrical modeling of carbon nanotube as an interconnect material, substituting Cu/low-k from quantum and classical considerations [13,14,15]. Recently, Srivastava *et al*. [16] have conducted exhaustive studies on single-wall carbon nanotube interconnect from one-dimensional fluid model and included electron-electron repulsive interaction and extended to multi-wall carbon nanotubes and bundles of single-wall carbon nanotubes [17]. Thermal effects in VLSI interconnect limits the current density and there have been problems of breakdown in carbon nanotubes due to resistive heating [18,19,20,21]. Huang *et al*. [22] have studied thermal transport and observed experimentally that the hottest spot is located at the center of the tube from where breakdown is initiated. The current transport is diffusive and not ballistic. Santini [23] has done exhaustive experimental studies on Joule heating-induced breakdown of carbon nanotube interconnects and attribute to the defect sites as also observed by Huang *et al*. [22]. Naeemi and Meindl [20,21] have studied temperature coefficient of resistance of single- and multi-wall carbon nanotube interconnects and related to various electron-phonon scattering mechanisms. Kitsuki *et al*. [24] and Yamada *et al*. [25] have explained experimental results of current induced breakdown in carbon nano fibers. In this work, we have examined the problem of Joule heating in carbon nanotube interconnects based on one-dimensional fluid model of electronic transport considering various scattering mechanisms and studied the temperature distribution across the length of the nanotube and scattering parameters. In Section 2 of the paper, we have developed one-dimensional fluid model incorporating thermal effects. In Section 3, temperature profiling across the length of the carbon nanotube is presented followed by the results and discussion. Conclusion is presented in Section 4.

## 2. Electrical and Thermal Transport Model

The conducting single-wall, multi-wall and bundle of single-wall carbon nanotube have been considered for possible replacement of Cu/low-k dielectric interconnects used in current CMOS technology depending upon the type of the interconnect, such as whether local or global. In the present work, we have considered Joule heating in a metallic single-wall carbon nanotube for better understanding of breakdown and also for analytical simplicity.

**Figure 1:**
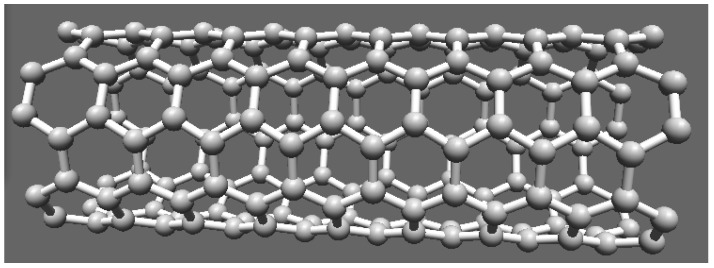
Single wall carbon nanotube.

In our earlier work [16], we have made modification in two-dimensional fluid model and included electron-electron repulsive interaction and considered distribution of conduction electrons on the lateral surface of single-wall carbon nanotube (SWCNT) cylindrical shell. The analysis reduces to semi-classical one-dimensional fluid model. The SWCNT is a two-dimensional graphene sheet rolled to form a cylindrical nanotube of infinitesimally thin layer. Thus, conduction electrons are distributed on the lateral surface of the SWCNT and motion of electrons is confined to the surface. As the diameter of CNT is very small we can also assume that electron is confined in one-dimensional space. We can assume that cloud of electrons is moving across the surface of the nanotube. Figure 1 shows a structure of a single wall carbon nanotube.

The motion of electrons across the SWCNT in a 1D fluid model can be described by the following equation [16,17],

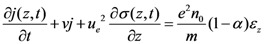
(1)
where *j* is the current density, *m* is effective mass of an electron, *n*_0_ is equilibrium three-dimensional electron density and σ is conductivity. Electron-electron repulsion factor is described by α. The thermodynamic speed of sound is *u*_e_; *e* is electronic charge, *ε*_z_ is electric field and *v* is relaxation frequency of electron. The Equation (1) describes the relation between an electric field and the current density.

We have modified the relaxation frequency (*v*) in Equation (1) considering the effective mean free path as follows,

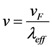
(2)


In Equation (2), *v*_F_ is Fermi velocity of electron and *λ_eff_* is effective mean free path of an electron which accounts for acoustic and optical phonon scattering mechanism responsible for increased resistance of the interconnect. The spontaneous scattering length for emitting an optical phonon (*λ_op_*) and acoustic phonon (*λ_ac_*) can be estimated as follows [26],

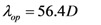
(3)

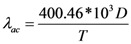
(4)


These two scattering lengths depend on diameter of the carbon nanotube (*D*) and surrounding temperature (*T*). The scattering length due to optical phonon absorption, *λ_op_*_,*abs*_ has been modeled by the following equation [19],

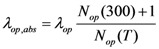
(5)


In Equation (5), *N_op_* describes the optical phonon occupation which can be calculated from Bose-Einstein statistics given by,

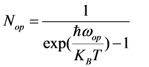
(6)
where 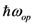 is optical phonon energy and its typical value varies from 0.16 eV to 0.20 eV [19]. In our work, we have taken this value as 0.16 eV. Occupancy function of optical phonon increases as temperature increases which eventually decreases optical phonon absorption component (*λ_op,abs_*) of electron mean free path. Optical phonon emission process has two components, one is for the absorbed energy (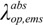) and another (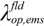) is for the electric field across the SWCNT length. Both of these components are expressed as follows [19],

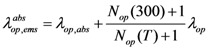
(7)

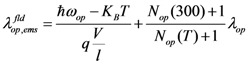
(8)


In Equations (5), (7) and (8), *N_op_*(300) is the value of phonon occupation function at 300 K. *K*_B_ in Equation (8) is the Boltzmann constant. Carrier scattering path due to optical emission under the influence of electric field (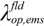) depends on both the electric field (*V*/*l*) and temperature (*T*). The effective mean free path can now be calculated by Matthiessen’s rule as follows,

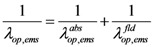
(9)

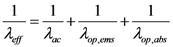
(10)


Assuming SWCNT as a good conductor and considering only one-dimensional flow we can deduce the current density from Equation (1) which is given by,

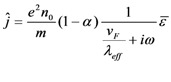
(11)
where ω is the angular frequency. The current density equation relating the frequency dependence conductivity of the SWCNTs, *σ*(*ω*) is expressed by,

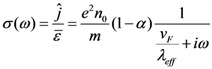
(12)
*σ*(*ω*) is also called as dynamic conductivity of SWCNT. For metallic CNT, semi-classical axial conductivity is given by [27],

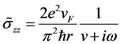
(13)


**Figure 2:**
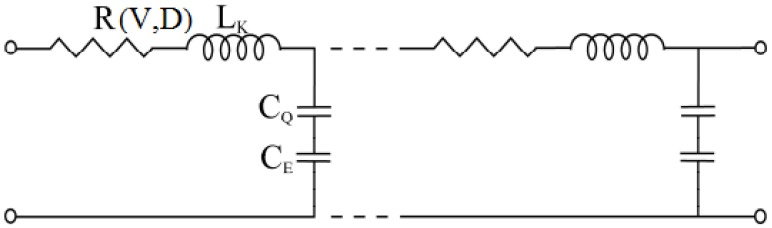
Transmission line model of SWCNT interconnect.

Combining Equations (12) and (13), we obtain,

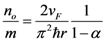
(14)
where *r* is the radius of SWCNT. Equation (14) relates dynamic conductivity with the semi-classical conductivity. We have assumed that SWCNT is metallic and placed above a perfectly conducting plane and also assumed that quasi-TEM electromagnetic wave is propagating through the SWCNT. The electric field can be expressed as [16],

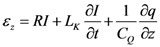
(15)
where *R* is the resistance in per unit length, *L*_K_ is kinetic inductance in per unit length and *C*_Q_ is quantum capacitance in per unit length. The SWCNT interconnect can then be better explained as a transmission line shown in Figure 2 where its parameters are described by following equations,

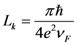
(16)

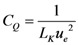
(17)

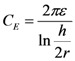
(18)

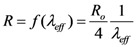
(19)

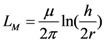
(20)

In Equation (18), *h* is the height of SWCNT over the perfectly conducting plane and *R*_0_ is quantum resistance taken as 12.906 kΩ and *L*_M_ is described by the Equation (20) as the magnetic inductance.

In thermal modeling of SWCNT interconnect material, *R*(*V*, *D*) is the voltage (*V*) and diameter (*D*) dependent critical parameter which directly depends on the effective mean free path (*λ_eff_*). Increase in temperature of interconnect triggers various scattering processes which result in increase of the resistance. This increased resistance limits the current density and eventually limits heat generation. Consequently, an iterative solution is needed to find out the actual converged value for resistance instead of using a direct equation.

## 3. Results and Discussion

Temperature profiles along the SWCNT length can be numerically solved from steady state heat equation given by Carslaw and Jaeger [28],

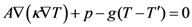
(21)
where κ is the heat conductivity, *A* is the Cross sectional area (*π* × *Diameter* × *Thickness*), *T'* is ambient temperature, *g* is thermal conductivity of the substrate and *p* is heating source power. In calculation, the terminal temperatures of SWCNT are assumed to be at 350 K which is the normal operating temperature of a typical bulk semiconductor. Therefore, in Equation (21), *T'* = 350 K and *g* = 0.15 Wm^−1^ K^−1^, have been used [19]. The standard feature size of the CNT has been used so that results can be compared with the findings of other reported work. CNT diameter, D has been taken as 1 nm which is a typical value. Resistance of SWCNT at different biasing voltages and lengths has been calculated by numerically solving Equation (21). Since, thermal conductivity (κ) is almost constant within 350 K to 800 K [19], we have taken thermal conductivity constant at a particular bias voltage. The steps in calculation of resistance are described as follows assuming ambient temperature remains constant as that of the CNT across its length.

1. Consider differential length of CNT and calculate mean free path as well as differential resistance for differential element using Equations (10) and (19). Estimate total resistance of SWCNT by summing all differential resistances.
2. Calculate current from Equation *I* = *V*/(*R* + *R*_c_), where *R*_c_ is the contact resistance 30 KΩ [19].
3. Calculate *I*^2^*R* per unit length for heat generation and then use Equation (21) to get temperature profile over the CNT length.
4. Use current temperature profile as the initial temperature for next iteration. Repeat steps 1 to 4 until convergence is obtained.

**Figure 3:**
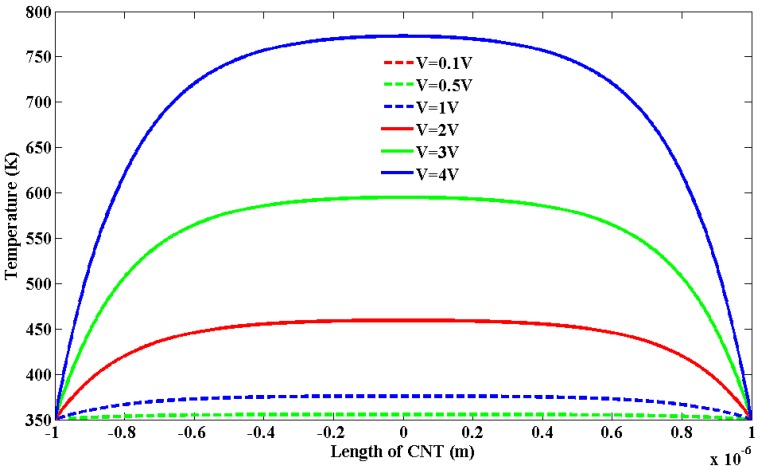
Temperature profile of SWCNT of 2 µm length.

Figure 3 shows the temperature profile over the length of SWCNT of 2 µm length. At different bias voltages, the temperature profile varies over the length. At biasing voltage above 3 V, the temperature reaches close to the breakdown temperature of 873 K [19]. From Figure 3, it can be inferred that biasing voltage less than 4 V is not sufficient enough for causing the breakdown of SWCNT. It is to be mentioned that for low biasing, the iteration requires less than 5 iterations to converge. On the other hand, high bias voltage more than 4 V, calculation takes more iteration to converge.

**Figure 4:**
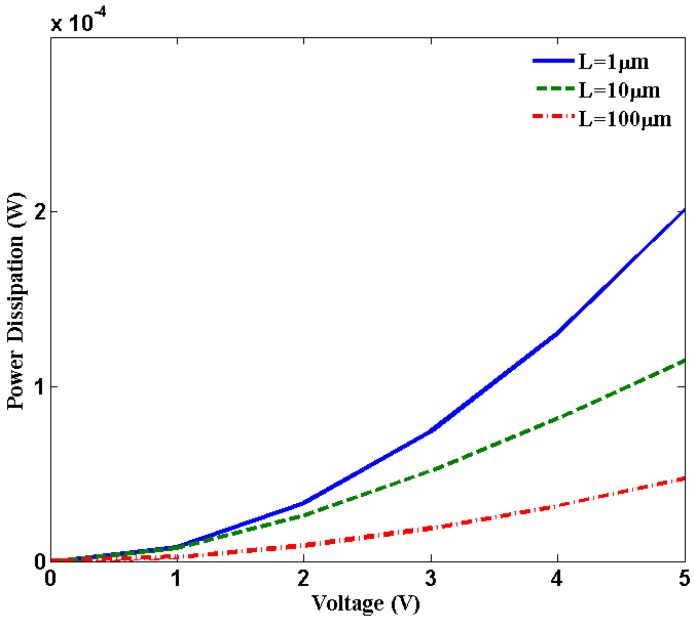
Power dissipation due to Joule heating along the SWCNT length.

Figure 4 shows power dissipation *versus* bias voltage. For 4 V biasing voltage, Joule heating power is close to 0.13 mW. As a matter of fact, at a certain bias voltage enough heat will be produced that will be sufficient for inducing breakdown in SWCNT at the mid-point. Higher biasing voltage increases optical phonon emission induced by the higher electric field. According to the Equation (8), electron scattering length 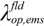 decreases with increase in bias voltage which contributes to decrease in effective mean free path (*λ_eff_*). The total resistance increases with decreasing effective scattering length. This increase in resistance contributes to increase in Joule heating.

**Figure 5:**
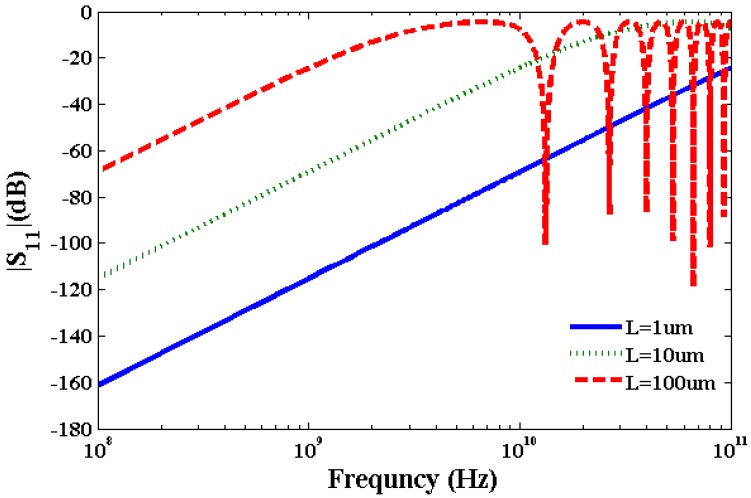
Plot of S_11_ parameter of SWCNT interconnects at 0.1 V bias voltage.

**Figure 6:**
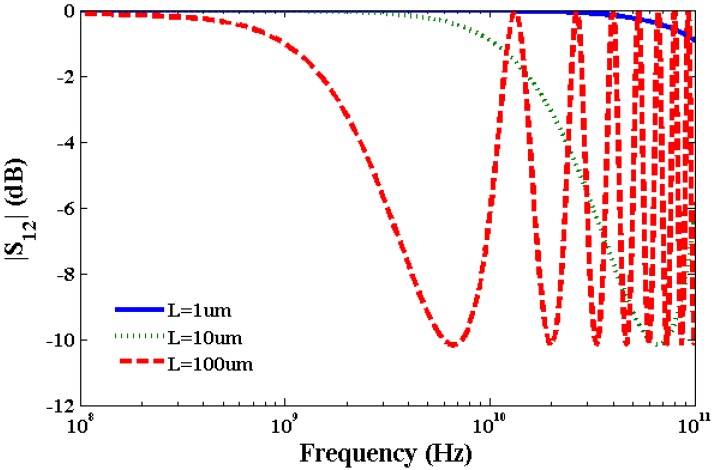
Plot of S_12_ parameter of SWCNT interconnects at 0.1 V bias voltage.

Using Equations (16)–(18), we have calculated kinetic inductance, *L*_K_ = 3.6 nH/µm, quantum capacitance, *C*_Q_ = 90 aF/µm and electrostatic capacitance *C*_E_ = 70 aF/µm of SWCNT transmission line to study S-parameters. Typically, a SWCNT diameter is ~1 nm and oxide thickness over which SWCNT is deposited is ~100 Å. The calculated value of *L*_M_ ~1 pH/μm which is very small compared to the value of *L*_K_. The calculated kinetic inductance is consistent with the value calculated in [29] for metallic CNTs. It is apparent from Equations (16)–(20) that inductance and capacitance are constant for a specific SWCNT with a given length and diameter. On the other hand, resistance of SWCNT interconnect is a function of bias voltage, SWCNT diameter, length, and temperature which influences scattering parameters. Scattering Parameter S_11_ is the ratio of power reflected from the transmission line to the incident power. Scattering Parameter S_12_ is the ratio of power transmitted through the transmission line to the incident power. Two port network parameters S_11_ and S_12_ have been calculated considering lumped elements and normalized by 50 ohm impedance. Although transmission line is a distributed device, we have used lumped element model to calculate S-parameters for the sake of efficient computation. Figure 5, Figure 6 show plots of S_11_ and S_1__2_ parameters of SWCNT at 0.1 V bias voltage. At higher frequencies inductive and capacitive terms dominate over the resistive term which results in an oscillatory behavior of S-parameters as observed in Figure 5, Figure 6. Figure 5 for the S_11_ parameter exhibits an oscillatory behavior for 100 µm long interconnect above 10 GHz in the frequency range studied. Figure 6 for the S_12_ parameter shows an oscillatory behavior for both 10 µm and 100 µm long interconnects above 70 GHz and 7 GHz, respectively. However, the short interconnect of 1 µm length does not show any oscillatory behavior for S_11_ and S_12_ parameters.

Table 1 summarizes the comparison of results with that reported in [16] without considering the Joule heating and with the Joule heating induced scattering with this current model. We have considered the frequency band in which value of S_1__2_ parameter is within −3 dB of its maximum value at 0.1 V. It is noticeable from Table 1 that the bandwidth reduces considering Joule heating due to scattering in comparison to the bandwidth without Joule heating. The frequency band width for a interconnect decreases considering Joule heating induced scattering and increased resistance.

**Table 1:** S_1__2_ parameters of SWCNT.

Length of SWCNT (µm)	Band Width (GHz) without Scattering [16]	Band Width (GHz) with Scattering
1	1000	120
10	110	11
100	30	1.0

**Figure 7:**
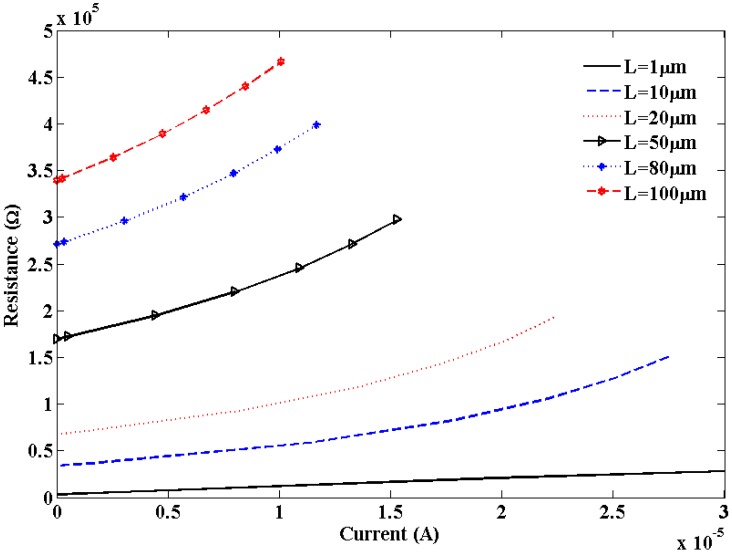
Plot of SWCNT resistance *versus* current.

**Figure 8:**
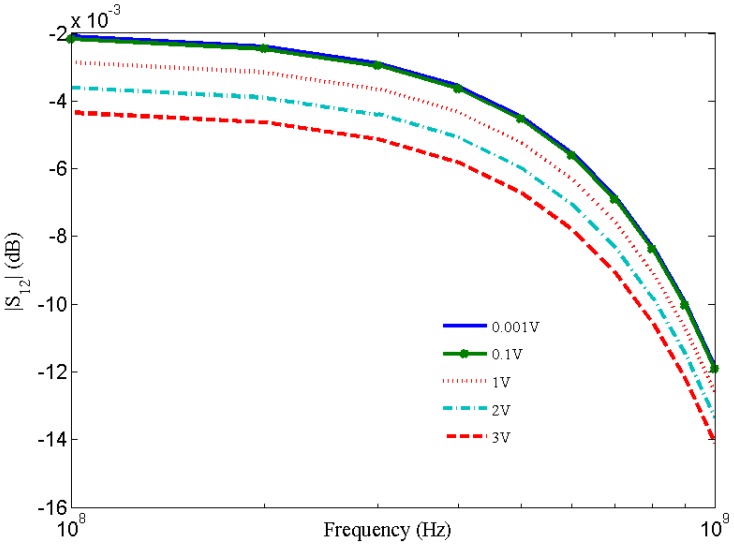
S_12_ parameter of SWCNT interconnects at different bias voltages.

Figure 7 shows resistance variation with the applied current for short local and global interconnects lengths. It is observed that breakdown in SWCNT occurs due to Joule heating which results in an infinite increase in resistance. This theoretical observation agrees with the experimental observation [30]. Figure 8 shows the dependence of S_12_ on bias voltages and shows an increase in S_12_ with increased bias voltage.

## 4. Conclusions

In this work, we have incorporated Joule heating induced phenomenon in 1D fluid model of CNT interconnects. We have studied scattering parameters of SWCNT for short, local and global interconnect lengths with different biasing voltages. We have observed that the bias voltage does not greatly affect the scattering parameters; on the other hand it significantly influences Joule heating. The breakdown shown in resistance *versus* current is clearly notable. The presented thermal model is very useful in experimental studies related to CNT integrated in nm- and sub-nm CMOS technology.
